# Effects of Whole-Body Vibration-Assisted Training on Lower Limb Blood Flow in Children With Myelomeningocele

**DOI:** 10.3389/fbioe.2021.601747

**Published:** 2021-02-10

**Authors:** Andrzej Szopa, Małgorzata Domagalska-Szopa, Andrzej Siwiec, Ilona Kwiecień-Czerwieniec

**Affiliations:** ^1^Department of Physiotherapy, Medical University of Silesia in Katowice, Katowice, Poland; ^2^Department of Medical Rehabilitation, Medical University of Silesia in Katowice, Sosnowiec, Poland; ^3^John Paul II Pediatric Center in Sosnowiec, Sosnowiec, Poland

**Keywords:** spina bifida, doppler ultrasound test, range of motion, whole-body vibration, myelomeningocele, lower limb

## Abstract

This study investigated the effectiveness of whole-body vibration (WBV) training incorporated into a conventional physiotherapy (PT) program (WBV-assisted training) in improving blood flow in the lower limbs and range of motion in the lower limb joints of children with myelomeningocele (MMC). A total of 31 children with MMC (7–15 years old) underwent a 6 weeks treatment program consisting of 2 weeks of conventional PT followed by 4 weeks of WBV-assisted training. The assessment comprised two parts: evaluation of lower limb joint range of motion and Doppler ultrasonography of the superficial femoral, popliteal, and anterior tibial arteries and was performed three times for each of the participants (at baseline, after 10 sessions of PT but before WBV-assisted training, and after 20 sessions of WBV-assisted training). Our results showed that WBV-assisted training significantly improved lower limb circulation in patients with MMC, increasing velocity and reducing resistivity in all tested arteries. Moreover, WBV-assisted training alleviated lower-extremity contractures, especially of the knee. Thus, WBV-assisted training is effective as an adjunctive rehabilitation program for improving functional mobility in children with MMC.

## Introduction

Spinal cord malformation is a congenital defect caused by the incomplete closure of one or more vertebral arches during the first few weeks of gestation (Sandler, 2010). Three types of malformation can be distinguished based on the severity (Rossi et al., 2004): spina bifida occulta (SB); meningocele (MC); and myelomeningocele (MMC), which is the most severe form and involves protrusion of the meninges, spinal cord, and nerve roots through an opening in the spine, resulting in partial or complete paralysis of the body below the malformation. MMC can affect the thoracic, lumbar, or sacral region of the spine, with malformations at a higher level associated with a greater degree of neurologic disorder. MMC most frequently involves the lumbosacral region. The primary clinical features in a child with lumbosacral MMC are flaccid paralysis (muscle weakness and wasting), decreased or absent tendon reflex and exteroceptive and/or proprioceptive sensation, and congenital deformities (Vinck et al., 2010).

Although the symptoms of MMC vary widely, they usually include defects in the innervation of the lower limb and spinal muscles below the spinal cord injury and secondary effects such as lower limb paralysis and paresis, as well as spine and lower limb joint deformities that restrict movement or cause disability. Compared with their able-bodied peers, adolescents, and young adults with MMC have a significantly lower level of daily physical activity, lower aerobic fitness, and higher body fat percentage (Buffart et al., 2008b), which are the most serious risk factors for the development of cardiovascular disease later in life and are observed more frequently in MMC patients than in the general population (Buffart et al., 2008a,b).

There is limited information on the factors contributing to increased cardiovascular disease risk in the MMC population. One study investigating cardiovascular autonomic nervous system functioning in children and young adult patients with MMC found that those who used a wheelchair showed lower spontaneous baroreflex sensitivity than their healthy counterparts (Leonardi-Figueiredo et al., 2019). This was explained as an effect of lower limb hypomobility and muscle pump inefficiency or of peripheral vascular remodeling due to low orthostatic stress, even in the recumbent position (Leonardi-Figueiredo et al., 2019). These findings were partly confirmed by our recent work showing that children and adolescents with MMC tend to develop venous insufficiency, with increased duration of retrograde flow at proximal and peripheral locations in the great saphenous vein compared with their normally developing peers (Domagalska-Szopa et al., 2020). The prevalence and severity of venous insufficiency in these children were closely associated with the clinical severity of MMC, a greater deficit in thigh and leg muscle strength and lower functional independence (Domagalska-Szopa et al., 2020). Vascular remodeling in the lower extremities and alterations in the vascular characteristics of the arterial circulation (e.g., reduced diameter and blood flow and increased shear stress) have been reported in adults with SB (Boot et al., 2003). These are the manifestations—which are associated with a sedentary lifestyle—along with hypomobility of lower limbs, dysfunction of the venous muscle pump (due to muscle paralysis), and disorders of cardiovascular autonomic control predispose children with MMC to severe vasomotor changes and blood circulation disorders.

Children with MMC can have a variety of congenital orthopedic deformities (“clubfoot”), including calcaneovalgus, equinovarus, and vertical talus, as well as hip dislocation and congenital scoliosis (Sandler, 2010). Acquired musculoskeletal deformities are the result of imbalanced muscle development, postural effects of gravity, and growth, affecting lower limb muscle and promoting knee flexion contracture (Rossi et al., 2004; Vinck et al., 2010). These can impair the functional abilities of individuals with MMC, especially transfer and walking (Vinck et al., 2010). Therefore, preventing muscle contracture or increasing both passive and active motion of lower limb joints is the basic goal of MMC treatment programs. The management of MMC includes physiotherapy (PT) (e.g., Vojta or proprioceptive neuromuscular facilitation), manual therapy, hydrotherapy, and massage to manage contracture and deformations and improve muscle strength, motor activity, functioning, and self-care (Schoenmakers et al., 2009; Webb, 2009; Tan et al., 2017).

There has recently been considerable interest in using whole-body vibration (WBV) training with a rotary-type platform and high-frequency/low-amplitude vibratory input to achieve therapeutic goals in the MMC population. One study reported a positive effect on bone mineral density (Emara, 2014), whereas another showed improvement in motor function (Stark et al., 2015) in children with MMC treated with WBV. Given the broad spectrum of symptoms in MMC, including lower limb muscle paralysis/paresis/contracture, vasomotor changes, and peripheral blood circulation disorders, rehabilitation programs for MMC patients should focus on muscle activation and vascular system improvement. Axial vibration induces reflexive muscle contraction in subjects who cannot evoke voluntary contractions, such as those with MMC (Christova et al., 2010). Thus, WBV training in conjunction with conventional PT can potentially provide additional benefits to these patients.

Although it was demonstrated that WBV could alter blood flow in the legs of healthy young adults (Kerschan-Schindl et al., 2001; Lythgo et al., 2009) and increase blood flow and activating muscles in the legs of patients with spinal cord injury (Herrero et al., 2011), the effectiveness of vibration stimulation in improving blood flow in the lower limbs and reducing hip and knee contracture in children with MMC is unknown. The purpose of this study was to investigate the effect of WBV training used in conjunction with conventional PT (i.e., WBV-assisted training) on blood flow in the lower limbs and the range of motion (ROM) of lower limb joints in children with MMC. We hypothesized that WBV-assisted training would increase peripheral blood flow below the spinal cord injury and improve the passive motion of the hips and knees in this group.

## Materials and Methods

### Patients

The study design, protocol, and consent forms were in accordance with the Code of Ethics of the World Medical Association (Declaration of Helsinki) for experiments involving humans. The Ethics Committee of the Medical University of Silesia approved the study (PCN/0022/KB1/64/I/19). Before the study, interviews were conducted with the children and their parents to explain the purpose, procedures, and potential benefits and risks of the study. Informed written consent was obtained from the parents before participant enrollment in the study.

The inclusion criteria were as follows: (1) a diagnosis of MMC; (2) age > 7 years (to minimize the incidence of unstable blood flow parameters); (3) ability to follow verbal directions; and (4) no complications beyond those common for MMC (e.g., hydrocephalus, clubfeet, and Arnold–Chiari malformation). Patients were ineligible for the study if they had any of the following: (1) a history of recent surgery (at least 1 year post-orthopedic surgery); (2) other acute or chronic diseases not related to MMC; (3) an allergy to the gel used for Doppler ultrasound examination; (4) no possibility of accessing the area of blood vessels examined with the handheld ultrasound device; (5) mental disorder; and (6) a history of epilepsy.

Participants with MMC were recruited through local pediatric rehabilitation centers and special interest support groups. Participant characteristics are listed in Table 1. Sex, age, body weight, height, body mass index (BMI), lower leg length, and leg circuits (LCs) of each participant were recorded. Height was measured using a height scale, and weight was measured using an electronic weighing scale (Tanita Corp, Tokyo, Japan). Height was measured in the standing position for participants without lower-extremity contracture who were able to remain standing. Those who could not stand upright and had contracture were measured in the seated and supine positions, and segmental measurements obtained from hip to knee and from knee to the sole of the foot were added to sitting height. BMI was calculated using the formula BMI = body weight (kilograms)/height^2^ (meters), and BMI percentile was determined using BMI Percentile Calculator for Child and Teen. The lower leg length was measured from the anterior superior iliac spine to the medial malleolus. The LC was measured using a tape measure at two levels of the leg, LC1 (the widest LC) and LC2 (at the malleolus).

**TABLE 1:** Accelerometer placement and stabilization for assessment of the range of motion (ROM).

Test—assessment Position of the accelerometer. Stabilization	Graphic description
T1 Deficit of hip flexion. Supine, both lower limbs extended resting on a support. 10 cm proximal to the lateral epicondyle femur, parallel to the long axis of the femur. Pelvis	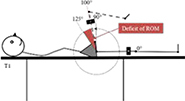
T2 Deficit of hip extension. Prone, both lower limbs extended resting on a support. 10 cm proximal to the lateral epicondyle femur, parallel to the long axis of the femur. Pelvis	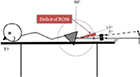
T3 Deficit of knee extension. Supine, hip of tested leg in neutral position in all planes and knee flexed 90^o^, off the table with the ankle in a neutral position contralateral extended leg resting on support. Anterior, 5 cm proximal to the lateral malleolus, parallel to the long axis of the fibula. Pelvis and thigh	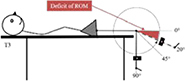
T4 Deficit of knee Flexion. Prone, hip of tested leg in neutral position in all planes and knee extended resting on a support. Anterior, 5 cm proximal to the lateral malleolus, parallel to the long axis of the fibula. Pelvis and thigh	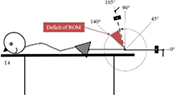
T5 and T6 Deficit of ankle dorsiflexion/plantarflexion (knee flexed). Supine, hip of tested leg in neutral position in all planes and knee flexed 90°, off the table with the ankle in a neutral position. Lateral, 10 cm distal to the lateral malleolus, parallel to long axis of metatarsal bones. Pelvis and thigh and leg	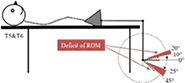

### Classification of Motor Function

The neurologic lesion level (NLL) of each participant was classified according to the area/level of MMC as follows: high lumbar, NLL = 1; low lumbar, NLL = 2; and sacral, NLL = 3. Motor function in line with each participant’s age was assessed and classified according to the revised and expanded version of the Gross Motor Function Classification System (GMFCS) (Palisano et al., 2008). Gross motor function was assessed using the Gross Motor Function Measure 88 score sheet (GMFM-88) in the following five domains: lying and rolling (17 items); sitting (20 items); crawling and kneeling (14 items); standing (13 items); and walking, running, and jumping (24 items) (Russell et al., 1989). The total score (ranging from 0 to 100) was calculated as the sum of scores for the five domains (Russell et al., 1989). GMFM-88 has been shown to be highly reliable and valid (with intraclass correlation coefficients of greater than 0.98 and 0.99, respectively) and may be used in clinical practice and research (Ko and Kim, 2013).

### Physical Examinations

The examination comprised two interrelated parts: (1) evaluation of ROM and (2) Doppler ultrasound examination of lower limb blood flow. The examinations were performed three times for each participant: at baseline (Pre-PT), after 10 sessions of PT and before WBV-assisted training (Pre-WBV), and after 20 sessions of WBV-assisted training (Post-WBV).

#### Range of Motion Assessment

Each participant underwent an examination of the ROM in the hip, knee, and ankle of each leg using an accelerometer. The positioning of the accelerometer and standardization procedures for all measurements are shown in Table 1. All participants were examined by the same experienced physiotherapist, who was blinded to the PT intervention program. For each test, the deficit in ROM was recorded. The accelerometric tests have been shown to be highly reliable [with the intraclass correlation coefficient (3.3) ranging from 0.79 to 0.95.] for use in the clinical practice and research in children with cerebral palsy (Szopa et al., 2014).

#### Blood Flow Analysis by Doppler Ultrasonography

After completing the ROM assessment, each participant was placed in the supine position on an examination table for blood flow measurement. Participants rested for 10 min to stabilize blood flow in the legs before ultrasound measurements. The examination was performed in a single experimental room between 14:00 and 17:00 h with the temperature maintained between 22 and 24°C by an experienced technician trained in vascular disease ultrasonography, which was blinded to the PT intervention program and to all previous data. Duplex scanning was performed separately on both lower limbs in three arteries: A1 [superficial femoral artery (SFA), a proximal section, approximately 2 cm before bifurcation of the common femoral artery in the deep and superficial femoral artery near the saphenofemoral junction]; A2 [popliteal artery (POPA), in the central portion of the popliteal fossa]; and A3 [anterior tibial artery (ATA), a distal section near the ankle level] (Yazici et al., 2007). The scanning site for subsequent measurements was marked with a permanent marker. Each image recorded by duplex scanning corresponded to a period of 4 s and included three to six beats. The mean of these beats was used to determine the following parameters (Yazici et al., 2007): peak systolic velocity (PSV), end-diastolic velocity (EDV), resistivity index (RI), and pulsatility index (PI). PSV (centimeters/second) is the highest systolic flow velocity in the SFA and POPA; EDV is the end-diastolic flow velocity in the SFA and POPA; and RI and PI are typically used to assess the resistance in a vascular system. RI reflects the resistance to blood flow caused by the microvascular bed distal to the site of measurement, whereas PI is a measure of pulsatile blood flow and is calculated from the maximum, minimum, and mean Doppler frequency shifts during a defined cardiac cycle.

The measurements were performed with the patient placed on an examination table with an adjustable height in the reverse Trendelenburg position (i.e., supine position with head and torso elevated on a wedge). The patient’s hips were generally abducted and externally rotated, and the knees were flexed like frog legs to facilitate the approach to the popliteal artery in the popliteal fossa and the posterior tibial artery in the medial calf (Yazici et al., 2007). The lower limb position upon external rotation of the hip and slight flexion of the knee helped decrease muscle tension and was suitable for exposing the arteries in the medial thigh and posterior knee. Rectangular support rollers were placed under the head, shoulders, buttocks, and feet to keep the entire lower extremity at a distance from the examination table.

All tests were carried out in triplex mode according to the recommendations of the Polish Society for Vascular Surgery and the Polish Society of Phlebology (Małek et al., 2014). Ultrasonography was performed using the SonoScape Expert 8 ultrasound machine with color flow imaging (Providian Medical Equipment, Highland Heights, OH, United States) and a linear probe with the Doppler effect (5–7.5 MHz L743).

### Physiotherapy Program and Whole-Body Vibration Training

**FIGURE 1:**
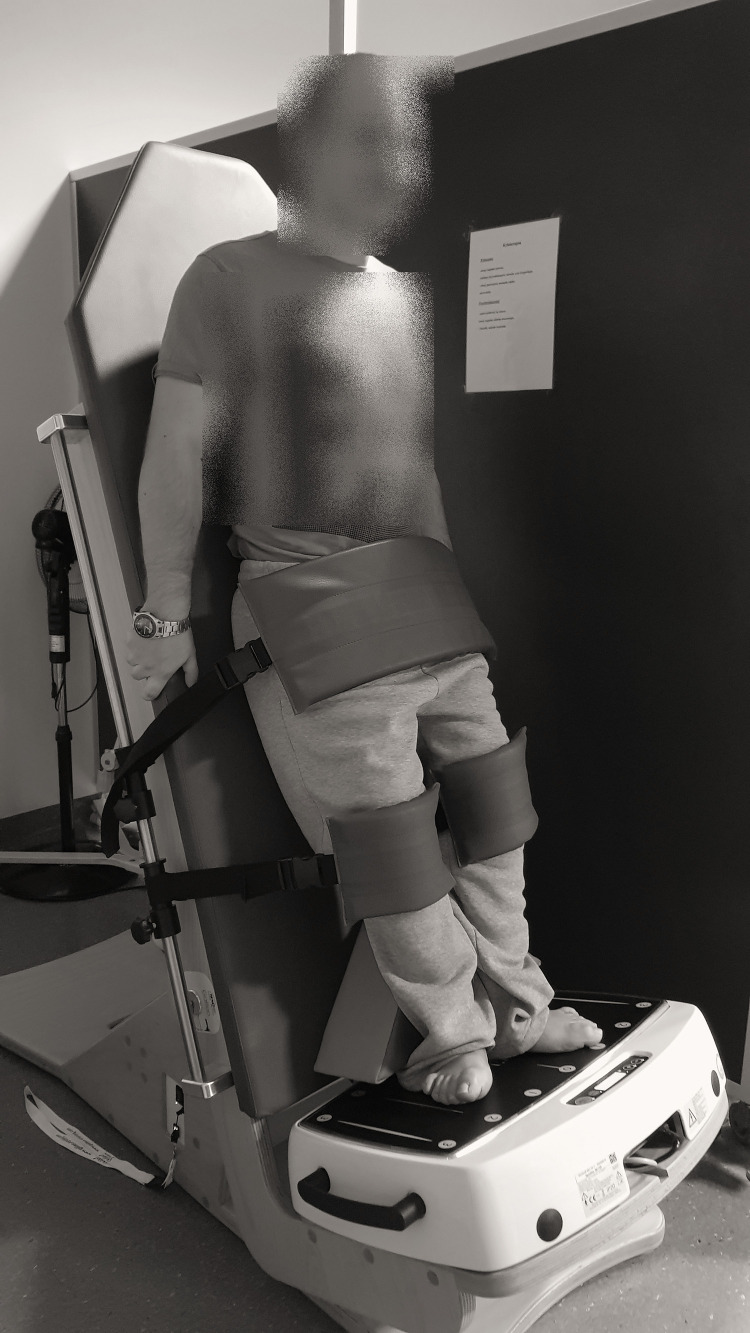
Placement of the participant on the tilt table during the application of the whole-body vibration training.

Participants underwent a 6 weeks treatment program at the local pediatric rehabilitation center. The PT program, which was conducted five times weekly for 6 successive weeks, consisted of 1 h of personalized exercises that included stimulation to maintain or improve muscle strength; stretching of tight muscles to prevent the development of contracture; correction of posture in the lying, sitting, and standing positions; exercises to improve balance and coordination; and standing positioning and gait training exercises using devices (e.g., standing frame, parapodium, and treadmill). After 2 weeks of conventional PT intervention, all participants received one WBV training session in conjunction with PT on the second half of each day of PT (5 days per week) for the following 4 weeks. WBV was applied *via* a vibrating platform constructed on a tilt table (TiltTable Galileo; Novotec Medical GmbH, Pforzheim, Germany) (Figure 1). This device is equipped with a platform that produces side-to-side alternating vertical sinusoidal vibrations around a fulcrum in the middle of the plate along with a tilt table, which is motor-driven and has an adjustable angle (0–90°) that makes it possible to perform WBV training with reduced body weight for patients who are unable to stand without support (Ruck et al., 2010).

Before WBV training, each participant was placed on the upper surface of the tilt table in a horizontal position (table tilt angle of 0°) with the feet supported approximately 8.0–20.0 cm apart and parallel to the axis defined by the habitual position of the lower limbs. Extra stabilization straps were placed on the knees and feet for complete fixation to the table and platform. The table was then tilted upward to the final position, which was individually set for each participant and mainly based on the angle of contracture of the knee. The final table tilt angle was estimated as complementary to the angle of affected knee contracture, such that the sum of table tilt and knee contracture angles was 90°. For example, if the angle of knee contracture was 45°, the angle of the table tilt was 45° (for a total of 90°). If there was no contracture in the knees, then the minimal knee flexion angle was 10°. Before the first WBV session, 3 min vibrations with a gradually increasing frequency from 12 to 25 Hz at a platform angle of 45° were administered to prepare the participant for the vibrations produced by the platform. Each WBV training session lasted 18 min and consisted of the following program: (1) 3 min of WBV; (2) 3 min of rest; (3) 3 min of WBV; (4) 3 min of rest; (5) 3 min of WBV; and (6) 3 min of rest. Thus, in one training session, participants were exposed to WBV for 9 min and rested for 9 min. The platform was set to produce a peak-to-peak displacement of 3 mm, and the frequency of vibration was set at 25 Hz with a peak acceleration of 0.3 × g (Rohlmann et al., 2014). The WBV training was adapted from a previously published observational study that used the same WBV system like ours to treat children with MMC (Emara, 2014). Kerschan-Schindl et al. (2001) reported that WBV training with the following parameters (26 Hz, 3 mm amplitude) effect on increase blood volume in the calf and thigh muscles with a significant increase in the mean blood flow velocity in the popliteal artery. WBV training sessions were conducted by a single experienced physiotherapist to ensure correct and accurate application. She was blinded to both physical examination results and USG Doppler test data.

### Statistical Analysis

Statistica v12.0 software (Statsoft, Tulsa, OK, United States) was used for statistical analyses. Descriptive statistics were computed for all data and are presented as the mean (standard deviation) or median (range) as appropriate. The Kolmogorov–Smirnov normality test was applied to the collected data, which showed significant differences from a normal distribution in all of raw ROM scores and for most of Doppler indices (all *p* < 0.05). Friedman’s rank test for dependent samples was used to compare three repeated measurements: Pre-PT, Pre-WBV, and Post-WBV. Differences across trials were considered statistically significant when the *P*-value was <0.05. As all participants completed the interventions, no adjustments were needed for dropouts. The Pearson’s correlation test was used to examine the relationship between the ROM deficit of lower limb joints and Doppler velocity (PSV and EDV) and resistivity and pulsatility indices (RI and PI, respectively), before and after conventional PT intervention and after WBV-assisted training. Spearman’s correlation test was used to examine the relationship between MMC and GMFCS scores and Doppler velocity, resistivity, and pulsatility before and after conventional PT intervention and after WBV-assisted training. Coefficients with a *P* < 0.05 were considered significant. Correlations were interpreted according to published guidelines as follows: *r* < 0.2, poor; *r* = 0.21–0.4, fair; *r* = 0.41–0.6, moderate; *r* = 0.61–0.8, good; and *r* = 0.81–1, very good (Altman, 1991).

## Results

### Characteristics of the Study Population

A total of 37 children diagnosed with MMC who met the inclusion criteria were sequentially enrolled in the study. Three patients could not be included because of a lack of access to the popliteal fossa by a handheld ultrasound device (POPA evaluation), and three others did not complete the study for reasons unrelated to WBV (i.e., there was no final examination). Thus, data for 31 participants (15 males and 16 females) with a mean age of 11.5 years (standard deviation, 3.6 years; range 7–15 years) were available for final analysis. Participants’ demographic characteristics (age, sex, weight, height, BMI, and BMI percentile), functional characteristics (GMFCS level and GMFM-88 score), and neurologic characteristics (NLL) are presented in Table 2. Among the participants, only 7 (23%) had normal BMI, 13 (42%) were overweight, and 11 (35%) were obese. The majority (48%) were level II GMFCS, whereas the others were level III (26%) and IV (26%). The most common NLL was the lumbar (high and low) region.

**TABLE 2:** Demographic, functional, and neurological characteristics of the children with MMC (MMC group).

Characteristics	MMC (*N* = 31)
Age (years), mean (*SD*)	11.5 (3.6)
Sex, boys, *N* (%)	15 (48)
Weight (kg), mean (*SD*)	48.3 (17.3)
Height (cm), mean (*SD*)	142.8 (14.7)
BMI (kg/m^2^), mean (*SD*)	22.9 (4.1)
BMI percentile, mean (*SD*)	86.4 (13.5)
**Functional characteristics GMFCS levels**	
I, *N* (%)	0 (0)
II, *N* (%)	15 (48)
III, *N* (%)	8 (26)
IV, *N* (%)	8 (26)
GMFM 88 D and E domain score%, mean (*SD*)	65.6 (13.9)
**Neurological characteristics lesion level**	
High lumbar, *N* (%)	13 (42)
Low lumbar, *N* (%)	11 (35)
Sacral, *N* (%)	7 (23)

### Clinical Assessment

#### Range of Motion Analysis

ROM measurements at baseline (Pre-PT) and before (Pre-WBV) and after (Post-WBV) intervention are shown in Table 3. There were no statistically significant differences between Pre-PT and Pre-WBV ROM values or between Pre-WBV and Post-WBV ROM values. Two weeks of conventional PT intervention significantly improved the ROM of the ankle and hip flexion and extension, whereas WBV-assisted training improved ROM in all lower limb joints, including knee extension.

**TABLE 3:** Deficit of the ROM of lower limb variable comparison at baseline (Pre-PT), after 10 sessions of PT and before WBV-assisted training (Pre-WBV), and after 20 sessions of WBV-assisted training (Post-WBV).

	Pre PT (*N* = 62)	Pre WBV (*N* = 62)	Post WBV (*N* = 62)	Statistical test	W
	Mean (95% CI)	Mean (95% CI)	Mean (95% CI)	*p*-values	
Hip flexion (°)	10.76 (8.31–13.20)	10.00 (7.58–12.41)	8.92 (6.62–11.21)	χ*^2^* = 62.48; **<0.001**^bc^	0.988
Hip extension (°)	31.90 (29.52–34.27)	31.68 (29.31–34.03)	30.71 (28.41–33.00)	χ*^2^* = 60.57; **<0.001**^bc^	0.985
Knee flexion (°)	8.18 (6.05–10.29)	7.79 (5.73–9.84)	6.58 (4.80–8.35)	χ*^2^* = 56.62; **<0.001**^bc^	0.983
Knee extension (°)	19.77 (16.85–22.69)	19.45 (16.57–22.32)	15.27 (12.42–18.12)	χ*^2^* = 96.08; **<0.001**^bc^	0.976
Ankle Dorsiflexion (°)	10.58 (6.83–14.32)	10.37 (6.70–14.04)	9.74 (6.28–13.20)	χ*^2^* = 30.14; **<0.001**^c^	0.998
Ankle Plantarflexion (°)	28.15 (22.12–34.16)	27.91 (21.93–33.89)	26.95 (21.11–32.78)	χ*^2^* = 41.60; **<0.001**^bc^	0.999

*Means (95% CI) of the outcome and statistical tests and p-values.*

*CI, confidence interval; χ^2^, Friedman test for dependent samples. Statistically significant differences are printed in bold. Effect size = Kendall’s W.*

*^*a*^Difference between Pre-PT and Pre-WBV.*

*^*b*^Difference between Pre-WBV and Post-WBV.*

*^*c*^Difference between Pre-PT and Post-WBV. Dunn–Bonferoni post hoc test.*

#### Analysis of Blood Flow

Pre-PT, Pre-WBC, and Post-WBV Doppler velocity indices (PSV and EDV) and Doppler resistivity and pulsatility indices (RI and PI, respectively), for the SFA, POPA, and ATA are presented in Table 4. After 2 weeks of conventional PT intervention (Pre-WBV), PSV, EDV, RI, and PI did not differ from the baseline measurements (Pre-PT). Both PSV and EDV were significantly higher in the Post-WBV assessment for all three tested arteries, and RI was significantly lower for the SFA and POPA compared with the Pre-WBC values, whereas no significant differences between Post- and Pre-WBV values of PI were observed for the POPA and ATA. After WBV-assisted training, there was an improvement in both velocity indices (PSV and EDV) and reductions in the resistivity indices (RI and PI) in all tested arteries of both lower limbs. The lack of normative values for PSV, EDV, RI, and PI for the pediatric population precludes an interpretation of the baseline values of both velocity indices (PSV and EDV) and resistivity indices (RI and PI) in the tested arteries.

**TABLE 4:** Doppler velocity index (PSV and EDV) and resistivity index (PI and RI) variable comparison at baseline (Pre-PT), after 10 sessions of PT and before WBV-assisted training (Pre-WBV), and after 20 sessions of WBV-assisted training (Post-WBV).

Artery	Parameter	Pre PT (*N* = 62)	Pre WBV (*n* = 62)	Post WBV (*n* = 62)	Statistical tests *p*-values	Effect size
		Mean (95% CI)	Mean (95% CI)	Mean (95% CI)		
SFA	PSV (cm/s)	69.76 (65.80–73.71)	71.81 (67.75–75.87)	74.94 (70.46–79.43)	χ*^2^* = 40.74; **<0.001^b^**^c^	0.836
	EDV (cm/s)	8.22 (7.33–9.10)	8.63 (7.63–9.63)	9.91 (9.16–10.65)	χ*^2^* = 22.60; **<0.001^bc^**	0.734
	PI (index)	3.21 (2.74–3.68)	5.72 (5.23–6.21)	4.97 (4.51–5.44)	χ*^2^* = 7; **0.030^bc^**	0.643
	RI (index)	0.88 (0.87–0.90)	0.87 (0.86–0.89)	0.86 (0.86–0.87)	χ*^2^* = 51.60; **<0.001^bc^**	0.654
POPA	PSV (cm/s)	58.85 (54.91–62.79)	60.05 (56.11–63.98)	65.51 (61.13–69.89)	χ*^2^* = 36.03; **<0.001^bc^**	0.872
	EDV (cm/s)	7.45 (6.76–8.14)	7.76 (7.01–8.52)	9.06 (8.20–9.94)	χ*^2^* = 52.62; **<0.001^bc^**	0.839
	PI (index)	8.58 (7.39–9.76)	8.08 (6.88–9.29)	7.82 (6.79–8.87)	χ*^2^* = 9.39; **0.009^c^**	0.702
	RI (index)	0.88 (0.87–0.88)	0.87 (0.86–0.88)	0.86 (0.85–0.87)	χ*^2^* = 18.42; **<0.001^bc^**	0.743
ATA	PSV (cm/s)	44.41 (41.35–47.47)	45.28 (42.13–48.45)	47.81 (44.31–51.29)	χ*^2^* = 33.14; **<0.001^bc^**	0.786
	EDV (cm/s)	5.36 (4.64–6.07)	5.52 (4.70–6.34)	6.38 (5.72–7.05)	χ*^2^* = 17.06; **<0.001^bc^**	0.727
	PI (index)	9.13 (7.62–10.64)	8.83 (7.28–10.38)	7.99 (6.91–9.08)	χ*^2^* = 10.26; **0.006^a^**	0.829
	RI (index)	0.88 (0.87–0.89)	0.88 (0.87–0.89)	0.86 (0.85–0.87)	χ*^2^* = 5.51; 0.063	0.615

*Means (95% CI) of the outcome and statistical tests and p-values.*

*Superficial femoral artery (SFA); popliteal artery (POPA); anterior tibial artery (ATA); peak systolic velocity (PSV); end-diastolic velocity (EDV); resistivity index (RI); pulsatility index (PI); CI, confidence interval; χ^2^, Friedman test for dependent samples. Statistically significant differences are printed in bold.*

*^*a*^Difference between Pre-PT and Pre-WBV.*

*^*b*^Difference between Pre-WBV and Post-WBV.*

*^*c*^Difference between Pre-PT and Post-WBV. Wilcoxon’s post hoc test.*

The correlations between deficits in lower limb joint ROM (Pearson’s correlation), MMC level, and GMFM-88 score (Spearman’s correlation) and Doppler velocity/resistivity/pulsatility indices before intervention are summarized in Table 5. Although statistically significant correlations were observed between some RI values and ROM deficit and GMFM-88 score, the correlation coefficients were mostly low (*R* = 0.21–0.40). PSV and baseline RI of the POPA and ATA were correlated with a neurologic deficit and functional limitations: PSV was negatively correlated, whereas RI was positively correlated with deficits in knee extension and GMFCS level.

**TABLE 5:** Pearson’s (*r*) and Spearman’s (*R*) correlation.

Doppler test		*r*						*R*		
		
		Hip flexion	Hip extension	Knee flexion	Knee extension	Ankle flexion	Ankle extension	GMFCS level	GMFM 88 score	NNL
SFA	PSV	0.10	0.11	0.14	0.07	–0.13	0.12	–0.02	–0.02	–0.09
	RI	–0.08	–0.01	0.05	0.30*	0.06	0.31*	–0.01	–0.09	–0.08
POPA	PSV	0.03	0.08	0.08	−0.30*	–0.06	0.08	−0.29*	0.30*	–0.10
	RI	–0.10	–0.23	0.14	–0.13	–0.07	0.11	0.28*	−0.35*	0.17
ATA	PSV	0.16	0.19	0.19	−0.30*	–0.08	–0.01	−0.30*	0.02	−0.31*
	RI	–0.04	–0.12	0.07	0.06	–0.11	0.30*	–0.01	0.09	0.05

*p < 0.05.

## Discussion

The present study investigated the effectiveness of WBV training applied in conjunction with conventional PT in improving blood flow in the lower limbs and ROM of lower limb joints in children with MMC. The study was designed based on the assumption that differences in Doppler indices and ROM measurements between baseline and after 2 weeks of conventional PT intervention reflect the effects of conventional PT intervention alone on blood flow in the lower limbs and limb muscle contracture, whereas differences in these values before and after WBV-assisted training reflect the effects of 4 weeks of PT combined with WBV training.

The major finding of this study was that conventional PT combined with WBV training significantly improved vascular properties in individuals with MMC, whereas PT alone did not. That is, the intervention significantly improved most parameters of blood flow in the lower limbs. Specifically, WBV training in conjunction with conventional PT enhanced flow velocity in all tested arteries of both lower limbs, as evidenced by the significant differences between Post- and Pre-WBV values of velocity indices (PSV and EDV) in the SFA, POPA, and ATA (all *P* < 0.05). Moreover, there were improvements in vascular resistance after the intervention, as demonstrated by the decreases in resistivity and pulsatility (RI and PI, respectively). Although there was a decreasing tendency in the values of all tested arteries, differences between Post- and Pre-WBV RI and PI values were only statistically significant for the SFA. While the characterization of blood flow in the lower limbs of children with MMC was not the aim of this study, our findings are important because they suggest that children with MMC have poor circulation in their legs. However, this is purely speculative, as there are no standard values for hemodynamic parameters in the pediatric population, and there is no consensus in the literature regarding normal values for blood flow velocity (PSV and EDV) and resistivity (RI and PI) in the SFA, POPA, and ATA. Extrapolation of the values from adults indicated that most Doppler waveforms in children with MMC could be classified as having a “damped” pattern (Salari et al., 2019)—i.e., systolic acceleration and PSV were reduced, whereas diastolic flow was increased (Boot et al., 2003), with a concomitant increase in blood flow resistivity in the arteries of the lower extremities. The likely explanation for this pattern is the disturbance in the development of the vascular system due to defects in the innervation of the lower limb muscles below the spinal cord injury in individuals with MMC and lower limb paralysis and paresis starting almost from birth (Boot et al., 2003; Salari et al., 2019). Adults with SB (as well as those with MMC) are more prone to these secondary complications of poor circulation because of their smaller blood vessels and reduced lower-extremity blood flow (Boot et al., 2003) compared with individuals without spinal cord dysfunction or with acquired spinal cord injury (Boot et al., 2003; Salari et al., 2019).

A previous investigation of children with SB showed that systematic and active PT intervention diminished lower limb contracture (Pauly and Cremer, 2013; Oliveira et al., 2014). In our study, conventional PT significantly improved hip and ankle ROM, but WBV-assisted training further increased the ROM in all lower limb joints while also reducing knee flexion contracture in the participants. Similar results were obtained using a neuromuscular training protocol based on WBV in children with SB. This is an important finding because knee flexion contracture is considered a severely limiting impairment in children with MMC (Stark et al., 2015), as an absence of the usual axial load on the legs can lead to degenerative symptoms such as loss of bone integrity, which predisposes the patient to fragility fractures (Boot et al., 2003; Apkon et al., 2008).

The management of muscle and tendon contractures and lower limb joint deformities in MMC patients mainly includes stretching to increase knee flexion or surgical correction. However, contracture management is associated with a significant risk of complications arising from congenital disabilities, which predisposes these patients to fractures (Salari et al., 2019). Surgical correction of joint contracture in children with MMC was associated with an increased risk of multiple osteoporosis-related fractures (Apkon et al., 2008); the frequency of fractures in pediatric patients with SB was reported to be 11–30% (Boot et al., 2003; Apkon et al., 2008; Okurowska-Zawada et al., 2009). WBV was shown to increase bone mineral density in children with MMC without exposing them to potential fractures caused by vigorous stretching during traditional PT (Emara, 2014).

The low but significant correlation between hip and knee contractures and blood flow velocity and resistivity observed in our cohort indicated that the reduction of hip and knee contractures by WBV directly translated into improved circulation in the lower limbs. The application of WBV using platforms with a tilt table has certain advantages over conventional PT. Firstly, external stimulation of the muscle and high-frequency vibration can induce reflexive muscle contraction. Secondly, WBV is performed with the patient’s body in the vertical position, which increases skeletal muscle pump efficiency and orthostatic stress. WBV training with high-frequency/low-amplitude vibration in healthy individuals has been shown to induce muscle activity (Marin and Rhea, 2010), increase blood flow (Kerschan-Schindl et al., 2001), and enhance vascular functioning (Lythgo et al., 2009). In adult patients with spinal cord injury, WBV training increased both muscle mass and blood flow in the leg (Herrero et al., 2011). However, as there have been no studies investigating the effect of WBV on blood flow in the lower limbs of children with MMC, we are unable to validate our findings through comparisons with other work. Nonetheless, our results support our hypothesis that WBV-assisted training can enhance peripheral blood circulation below the spinal cord injury and reduce contracture of lower limb joints in individuals with MMC.

### Study Limitation

There were certain limitations to our study. Firstly, as there are no established standards for Doppler ultrasound examination of lower limb blood flow in children, we adapted those from published observational studies that used this technique in other pediatric populations. Secondly, the small sample size of patients with MMC (*n* = 31) may have prevented the detection of stronger associations. Finally, we did not investigate the benefits of WBV training alone.

## Conclusion

The severity of spinal cord lesions in MMC patients is unpredictable, and there are many other symptoms (e.g., bedsores and peripheral blood circulation disorders) and comorbidities (e.g., osteopenia, osteoporosis, and obesity) that can negatively impact the health status of these patients; PT to reduce these symptoms plays an essential role in the management of MMC. Although the effectiveness of WBV alone and in conjunction with conventional PT should be addressed in a randomized controlled trial with larger sample size, our results demonstrate that applying WBV through a tilt table is safe, easy to perform, and well tolerated by children with MMC and is thus a promising adjunctive treatment approach to standard rehabilitation programs for severely motor-impaired children and adolescents such as individuals with MMC.

## Data Availability Statement

The raw data supporting the conclusions of this article will be made available by the authors, without undue reservation, to any qualified researcher.

## Ethics Statement

The studies involving human participants were reviewed and approved by The Ethical Committee of the Medical University of Silesia. Written informed consent to participate in this study was provided by the participants’ legal guardian/next of kin.

## Author Contributions

ASz and MD-S designed the study. ASz, MD-S, IK-C, and ASi performed the experiments. ASz and ASi analyzed the data. ASz and MD-S wrote the manuscript. All authors contributed to the article and approved the submitted version.

## Conflict of Interest

The authors declare that the research was conducted in the absence of any commercial or financial relationships that could be construed as a potential conflict of interest.

## References

[B1] Altman D. G. (1991). *Practical Statistics for Medical Research.* London: Chapman and Hall/CRC.

[B2] Apkon S. D. Fenton L. Coll J. R. (2008). Bone density in children with myelomeningocele. *Dev. Med. Child Neurol.* 51 63–67. 18811711 10.1111/j.1469-8749.2008.03102.x

[B3] Boot C. R. van Langen H. Hopman M. T. (2003). Arterial vascular properties in individuals with spina bifida. *Spinal Cord.* 41 242–246. 12669089 10.1038/sj.sc.3101429

[B4] Buffart L. M. Roebroeck M. E. Rol M. Stam H. J. van den Berg-Emons R. J. (2008a). Triad of physical activity, aerobic fitness and obesity in adolescents and young adults with myelomeningocele. *J. Rehabil. Med.* 40 70–75. 18176740 10.2340/16501977-0135

[B5] Buffart L. M. van den Berg-Emons R. J. Burdorf A. Janssen W. G. Stam H. J. Roebroeck M. E. (2008b). Cardiovascular disease risk factors and the relationships with physical activity, aerobic fitness, and body fat in adolescents and young adults with myelomeningocele. *Arch. Phys. Med. Rehabil.* 89 2167–2173. 10.1016/j.apmr.2008.04.015 18835477

[B6] Christova M. Rafolt D. Mayr W. Wilfling B. Gallasch E. (2010). Vibration stimulation during non-fatiguing tonic contraction induces outlasting neuroplastic effects. *J. Electromyogr. Kinesiol.* 20 627–635. 20363152 10.1016/j.jelekin.2010.03.001

[B7] Domagalska-Szopa M. Szopa A. Puchner M. Schreiber L. Siwiec A. Hagner-Derengowska M. (2020). Leg venous properties in children with myelomeningocele. *Front. Pediatr.* 8:531. 10.3389/fped.2020.00531 32984225 PMC7492545

[B8] Emara H. A. (2014). Effect of whole body vibration on bone mineral density in children with myelomeningocele. *Dent. Med Sci.* 13 47–51. 10.9790/0853-13424751

[B9] Herrero A. J. Menendez H. Gil L. Martin J. Martin T. Garcia-Lopez D. (2011). Effects of whole-body vibration on blood flow and neuromuscular activity in spinal cord injury. *Spinal Cord* 49 554–559. 10.1038/sc.2010.151 21042329

[B10] Kerschan-Schindl K. Grampp S. Henk C. Resch H. Preisinger E. Fialka-Moser V. (2001). Whole-body vibration exercise leads to alterations in muscle blood volume. *Clin. Physiol.* 21 377–382. 11380538 10.1046/j.1365-2281.2001.00335.x

[B11] Ko J. Kim M. (2013). Reliability and responsiveness of the gross motor function measure-88 in children with cerebral palsy. *Phys. Ther.* 93 393–400. 10.2522/ptj.20110374 23139425

[B12] Leonardi-Figueiredo M. M. de Souza H. C. D. Martins E. J. Squiaveto M. Mattiello-Sverzut A. C. (2019). Damaged cardiovascular autonomic control in wheelchair-using children and adolescents with myelomeningocele: a case-control study. *Braz. J. Phys. Ther.* 23 27–32. 10.1016/j.bjpt.2018.09.001 30243858 PMC6546909

[B13] Lythgo N. Eser P. de Groot P. Galea M. (2009). Whole-body vibration dosage alters leg blood flow. *Clin. Physiol. Funct. Imaging* 29 53–59. 19125731 10.1111/j.1475-097X.2008.00834.x

[B14] Małek G. Elwertowski M. Nowicki A. (2014). Standards of the polish ultrasound Society—Update. Ultrasound examination of the aorta and arteries of the lower extremities. *J. Ultrason.* 14 192–202. 26673404 10.15557/JoU.2014.0019 PMC4579697

[B15] Marin P. J. Rhea M. R. (2010). Effects of vibration training on muscle strength: a meta-analysis. *J. Strength Cond. Res.* 24 548–556. 20072044 10.1519/JSC.0b013e3181c09d22

[B16] Okurowska-Zawada B. Konstantynowicz J. Kułak W. Kaczmarski M. Piotrowska-Jastrzȩbska J. Sienkiewicz D. (2009). Assessment of risks factors for osteoporosis and fractures in children with menin- gomyelocele. *Adv. Med. Sci.* 54 247–252. 19919941 10.2478/v10039-009-0039-y

[B17] Oliveira A. Jácome C. Marques A. (2014). Physical fitness and exercise training on individuals with spina bifida: a systematic review. *Res. Dev. Disabil.* 35 1119–1136. 10.1016/j.ridd.2014.02.002 24612860

[B18] Palisano R. J. Rosenbaum P. Bartlett D. Livingston M. H. (2008). Content validity of the expanded and revised gross motor function classification system. *Dev. Med. Child Neurol.* 50 744–750. 10.1111/j.1469-8749.2008.03089 18834387

[B19] Pauly M. Cremer R. (2013). Levels of mobility in children and adolescents with spina bifida-clinical parameters predicting mobility and maintenance of these skills. *Eur. J. Pediatr. Surg.* 23 110–114. 10.1055/s-0032-1324689 23093438

[B20] Rohlmann A. Schmidt H. Gast U. Kutzner I. Damm P. Bergmann G. (2014). In vivo measurements of the effect of whole body vibration on spinal loads. *Eur Spine J.* 23 666–672. 10.1007/s00586-013-3087-8 24201510 PMC3940795

[B21] Rossi A. Biancheri R. Cama A. Piatelli G. Ravegnani M. Tortori-Donati P. (2004). Imaging in spine and spinal cord malformations. *Eur. J. Radiol.* 50 177–200. 15081131 10.1016/j.ejrad.2003.10.015

[B22] Ruck J. Chabot G. Rauch F. (2010). Vibration treatment in cerebral palsy: a randomized controlled pilot study. *J. Musculoskelet Neuronal. Interact.* 10 77–83. 20190383

[B23] Russell D. J. Rosenbaum P. L. Cadman D. T. Gowland C. Hardy S. Jarvis S. (1989). The gross motor function measure: a means to evaluate the effects of physical therapy. *Dev. Med. Child Neurol.* 31 341–352. 10.1111/j.1469-8749.1989.tb04003 2753238

[B24] Salari F. Golpayegani M. Habibi Z. Yaghoubi S. Anbarlouei M. Mehdizadeh M. (2019). Evaluation of lower extremities’ vascular characteristics in myelomeningocele patients: a case-control study. *Pediatric Neurosurg.* 54 324–328. 10.1159/000502403 31487737

[B25] Sandler A. D. (2010). Children with spina bifida: key clinical issues. *Pediatr. Clin. N. Am.* 57 879–892. 10.1016/j.pcl.2010.07.009 20883878

[B26] Schoenmakers M. A. de Groot J. F. Gorter J. W. Hillaert J. L. Helders P. J. Takken T. (2009). Muscle strength, aerobic capacity and physical activity in independent ambulating children with lumbosacral spina bifida. *Disabil. Rehabil.* 31 259–266. 10.1080/09638280801923235 18608426

[B27] Stark C. Hoyer-Kuhn H. K. Semler O. Hoebing L. Duran I. Cremer R. (2015). Neuromuscular training based on whole body vibration in children with spina bifida: a retrospective analysis of a new physiotherapy treatment program. *Childs Nerv. Syst.* 31 301–309. 10.1007/s00381-014-2577-2 25370032

[B28] Szopa A. Domagalska–Szopa M. Kidonì Z. Syczewska M. (2014). Quadriceps femoris spasticity in children with cerebral palsy: measurement with the pendulum test and relationship with gait abnormalities. *J. Neuroeng. Rehabil.* 11:166. 10.1186/1743-0003-11-166 25516151 PMC4277843

[B29] Tan J. L. Thomas N. M. Johnston L. M. (2017). Reproducibility of muscle strength testing for children with spina bifida. *Phys. Occup. Ther. Pediatrics* 37 362–373. 10.1080/01942638.2016.1244872 28026982

[B30] Vinck A. Nijhuis-van der Sanden M. W. Roeleveld N. J. Mullaart R. A. Rotteveel J. J. Maassen B. A. (2010). Motor profile and cognitive functioning in children with spina bifida. *Eur. J. Paed. Neurol.* 14 86–92. 10.1016/j.ejpn.2009.01.003 19237302

[B31] Webb T. S. (2009). Medical care of adults with spina bifida. *J. Pediatr. Rehabil. Med.* 2 3–11. 10.3233/PRM-2009-0058 21791790

[B32] Yazici B. Simsek E. Erdogmus B. Bahcebasi T. Aktas A. Buyukkaya R. (2007). Evaluation of the thyroid blood flow with Doppler ultrasonography in healthy school-aged children. *Eur. J. Radiol.* 63 286–289. 10.1016/j.ejrad.2007.01.036 17374471

